# Rationale and design of an interventional study of cross-sectoral, coordinated treatment of stroke patients with patient-orientated outcome measurement (StroCare)

**DOI:** 10.1186/s42466-021-00107-2

**Published:** 2021-02-02

**Authors:** David Leander Rimmele, Theresa Schrage, Christian Brettschneider, Alexander Engels, Christian Gerloff, Martin Härter, Michael Rosenkranz, Holger Schmidt, Levente Kriston, Götz Thomalla

**Affiliations:** 1 Department of Neurology, University Medical Center Hamburg-Eppendorf, Martinistr. 52, 20246 Hamburg, Germany; 2 Department of Medical Psychology, University Medical Center Hamburg-Eppendorf, Martinistr. 52, 20246 Hamburg, Germany; 3 Department of Health Economics and Health Care Research, University Medical Center Hamburg Eppendorf, Martinistraße 52, 20246 Hamburg, Germany; 4 Department of Neurology and Neurological Early Rehabilitation, Albertinen Krankenhaus, Süntelstraße 11A, 22457 Hamburg, Germany; 5 Department of Neurology, Elbe Klinik Stade, Bremervörderstraße 111, 21682 Stade, Germany; 6 Department of Neurology, University Medical Center Göttingen, Robert-Koch-Str. 40, 37075 Göttingen, Germany

**Keywords:** Stroke, Quality of life, Stroke nurse, Case management, Implementation, Controlled trial

## Abstract

**Introduction:**

Stroke has a long-term impact on functional status and quality of life in multiple health domains. A well-coordinated managed care program for stroke patients is crucial for ameliorating patients’ health and cost-efficient use of resources. The aim of this study is the implementation and evaluation of an optimised cross-sectoral, coordinated and managed care program for stroke patients bridging secondary and tertiary care.

**Methods:**

In this multi-center mixed method sequentially controlled intervention study, stroke patients with ischemic stroke (I63), transient ischemic attack and related syndromes (G45), or intracerebral haermorrhage (I64) will be invited to participate. For a 12-months period, 235 consecutive patients are expected to be enrolled and assigned standard of care treatment as an active control group. During the following 12 months, 235 consecutive patients will be enrolled and assigned to a post stroke intervention program. The StroCare intervention consists of repeated outpatient visits with specialized stroke teams, the implementation of a case manager, the use of an electronical tool for communication between acute care, rehabilitation facilities, and out-patient care, and the definition of individualized treatment targets. Patients will be followed up for 24 months. The primary outcome is health-related quality of life measured by the Patient-Reported Outcomes Measurement Information System 10-Question Short Form (PROMIS-10) at 12 months after the index event, i.e. stroke or TIA. For the qualitative survey of the implementation process, 21 patients in the intervention group will be interviewed after implementation of the interventions. In addition, 20 health care providers and staff members will be interviewed before and after implementation. Additionally, economic outcomes will be evaluated after 6 and 12 months.

**Perspective:**

The study will not only provide information about the tested intervention but is likely to be helpful for clinicians, suppliers of reimbursement, and researchers in implementing and evaluating complex interventions in stroke care in general. With this program, the health care system will have a reference model at its disposal for transfer to other regions and settings.

**Trial registration:**

The trial is registered at ClinicalTrials.gov (NCT04159324). Approval of the local ethics committee (Ethik-Kommission der Ärztekammer Hamburg, Niedersachsen, Schleswig-Holstein) has been obtained.

## Introduction

Stroke is the most common cause of acquired long-term disability [1]. Many stroke patients have to live with a reduced functionality and changes in quality of life [2]. Increased life expectancy with a higher risk of stroke events and improved treatment interventions lead to more long-term survivors. After diagnosis and initial acute care, in general, inpatient or outpatient rehabilitative care follows in specialized clinics before long-term outpatient guidance by general physicians and neurologists. As a result, optimizing early steps towards rehabilitative care and detecting and preventing imminent recurrences at an early stage are becoming increasingly important in stroke care.

At present patients, the process of finding a place for neurorehabilitation and achieving treatment guarantee by the health insurance is often time consuming and leads to delay in the initiation of effective neurorehabilitative treatment [3].

Moreover, outpatient care after rehabilitation is largely insufficient [4]. Often, no medically experienced contact person is available for patients after rehabilitation [4]. This can lead to a compromised adherence to medication, insufficiently controlled risk factors and more frequent recurrent events. A lack of information of either the patients or their caregivers is one of the important reasons why follow-up treatments are not performed [4]. In up to 50% of the cases, the treatment goals regarding vascular risk factors are not met [5]. This represents a worldwide problem which was already by the World Health Organization WHO [6]. Complications needing treatment including repeated hospitalisations pose a strain for patients as well as for the healthcare system [7]. Furthermore, optimal stroke treatment depends on a multidisciplinary involvement and cooperation between in-patient clinics, general practitioners and physio- and occupational therapists, which is often lacking [4].

In order to improve stroke care in Germany, specific programs have been developed and are being tested. The program STROKE-OWL focuses on providing “stroke-guides”, similar to a case-manager (https://stroke-owl.de). The program SANO intends to build an integrated (intersectoral and interprofessional) network for stroke patients [8].

Stroke patients´ impairment to their quality of life can be measured by patient-reported outcomes (PROMs) and is associated with stroke severity and comorbidities(D. L. [9]). The EPOS approach comprised the implementation of the International Consortium for Health Outcome Measurement (ICHOM) Standard set for Stroke in clinical routine (David Leander [10]) and repeatedly assessed quality of life in follow ups at different time points after stroke [11].

## Methods

### Aim of the study

The aim of the present study is to evaluate cross-sectoral, coordinated and evidence-based stroke care (StroCare) in three hospitals and five rehabilitation centers, with three objectives: a) to assess the intervention effects on outcomes (effectiveness); b) to explore the implementation process; and c) to investigate economic outcomes.

### Study description and study design

**Fig. 1:**
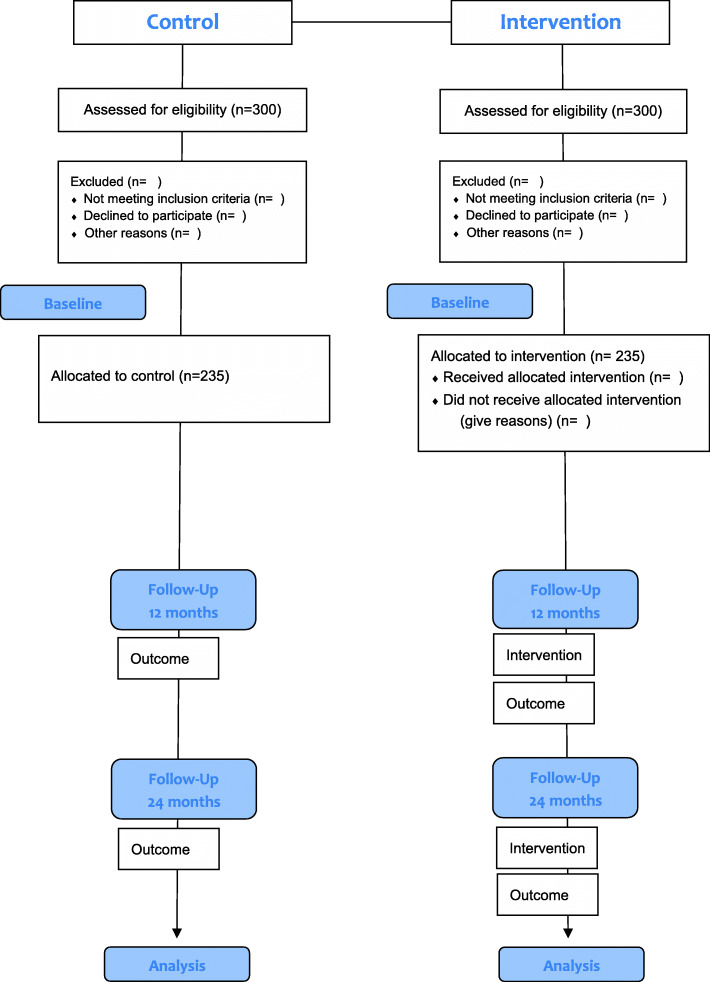
Exprected patient flow diagram for the assessment of effectiveness

The StroCare intervention will be evaluated in a multi-center mixed-methods sequentially controlled intervention study with a longitudinal design (Fig. 1). Onset is acute inpatient stroke treatment with subsequent neurological rehabilitation. Patients will be enrolled during their initial stay at the stroke unit for the control and intervention group during a 12 months period each, starting with the control group. Afterwards, the intervention will be deployed and enrolment in the intervention group will start. For individual patients, the intervention will cover a period of 24 months. The intervention effects on outcomes will be evaluated by a quantitative survey 12 and 24 months after stroke. In terms of the implementation process, qualitative interviews will be conducted with patients after the implementation of the interventions. Additionally, employees (doctors, nurses, and IT staff) involved in the study will be interviewed before and after the implementation of the interventions. Comparative analyses of health-care costs are planned 6 and 12 months after admission with admitted permission to insurance data files. Enrolment of patients in the control group started in January 2020. Scheduled duration of the entire study from first-patient-in to last-patient-out has been extended from 24 to 36 months due to the COVID-19 pandemic.

### Eligibility criteria

Patients will be included according to the following criteria: treatment at one of the participating acute clinics, diagnosis (ICD-10) of ischemic attack (I63), transient ischemic attack and related syndromes (G45), intracerebral haemorrhage (I64), insurance with the BARMER health insurance agency, sufficient mastery of German language and written informed consent. BARMER insurance membership is necessary due to its cooperation to defray the follow-up examination costs and to give access to patient data. Exclusion is based on the following criteria: premorbid score of mRS≥4, present diagnosis of artificial respiration (Z99.1), dementia (F00.x., F01.x. or G30.x) or aphasia (R47), substantially impaired communication capacity due to aphasia or dementia and admission to a nursing home following the acute treatment.

Patients will be screened by study nurses via electronic health record for diagnosis and insurance company after admission to the stroke unit. They will not be involved in patients´ treatment.

### Sample size calculation

In order to assess the effectiveness of the StroCare intervention, the two prospective groups will include all patients insured with the health insurance BARMER who will be hospitalised in one of the three participating clinics over a period of 24 months for stroke treatment and have given their written consent. Previous studies showed moderate to large effects of similar interventions on health-related quality of life with standardized mean differences (Cohen’s *d*) between treatment and control groups ranging from 0.2 to 0.6 [8, 12]. We aim to identify a moderate effect (Cohen’s *d*=0.3) of the intervention on patient’s experienced health-related quality of life. With a power of 80% and an alpha error of 0.025 (adjusted for two primary endpoints, the two subscales of the PROMIS-10), a sample size of *N*=213 patients per group (426 in total) will be needed. Accounting for an expected dropout rate of 10% results in a necessary sample size of 235 per group (470 in total). In the past few years, approximately 600 patients insured with BARMER with a diagnosis of stroke were treated annually in the three participating clinics. In the planned time frame of 24 months we thus expect to enroll the planned number of 426 patients if 39% of 1200 participants participate.

For the qualitative interviews it is indicated that on average 12 interviews are sufficient to reach theoretical saturation [13]. Thus, the planned sample size of a maximum of 21 patients enrolled in the intervention group and 20 employees of different departments (doctors, nurses, and IT staff) represents a meaningful basis for the investigation of the experience gained during the implementation.

### Arms and interventions

#### StroCare intervention

**Fig. 2:**
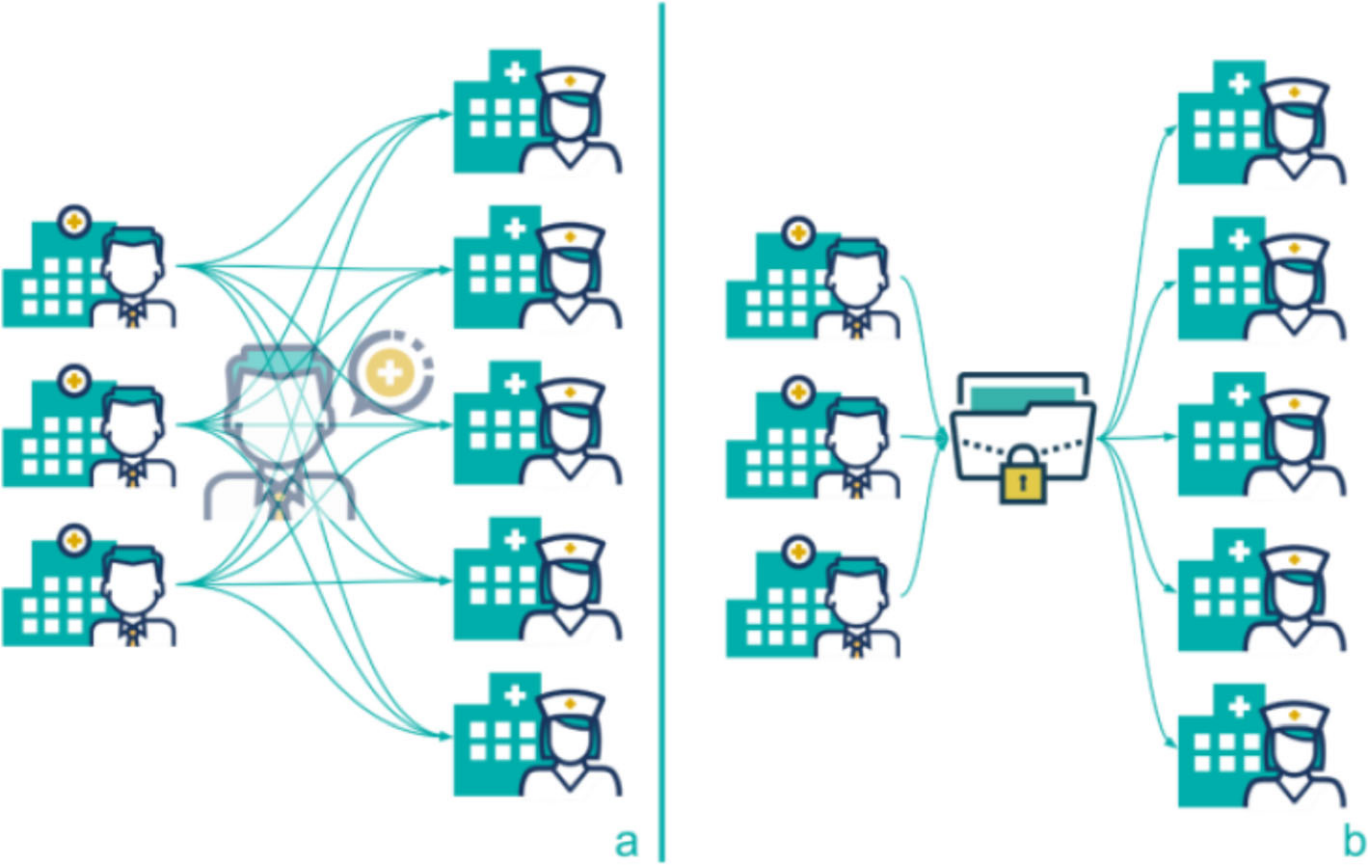
Placement allocation in usual care and in the StroCare intervention. **a** usual care: medical staff inquires separately by telephone for vacancies in rehabilitation clinics. **b** StroCare: all inquiries are forwarded to the rehabilitation clinics and back via electronical platform

The key element will be, on the one hand, improved cooperation between the various health care providers across sectoral boundaries (acute hospital, neurological rehabilitation, outpatient aftercare) and a health insurance company. In addition, optimized secondary prevention, risk factor control and risk adjustment in accordance with guidelines is going to be achieved by establishing a specific follow-up care offer at all participating neurological clinics. The StroCare intervention is a multicomponent procedure aimed at different persons and points of stroke care. Its main components are:

- *Primary contact -* A study nurse of the department of Neurology of the UKE will contact the patient during primary acute stroke treatment. This nurse will be the responsible contact person for the patient for all medical questions for the next two years. The outpatient managed care will also be organised by the stroke nurse. Every three months for the following two years, the responsible stroke nurse maintains contact to the patient via telephone and every six months, arranges a consultation with the patient in the outpatient clinic including a check-up (ECG, blood samples) and a physical examination.
- *Outpatient managed care -* To achieve an well-structured outpatient care and to ensure regular follow-up appointments, the patients will be scheduled for a check-up and physical examination every six months at the respective acute clinic. The follow-up treatment will be performed by a study nurse and a neurologist experienced in stroke care and will comprise a neurological examination, carotid and intracranial ultrasound, evaluation of risk factors, and planning of further treatment.
- *Case Management -* In addition to establishing contact with a stroke nurse, each patient is assigned a case manager. The case manager acts on behalf of the health insurance company and the first contact is made during acute inpatient care. The case manager supports the patient regarding organisational issues, whereby the individual social situation is taken into account. In addition to care-relevant issues from the statutory health insurance system (like a request for classification of the degree of care), issues concerning the social nursing care insurance will also be addressed. The family, professional and financial situation, the housing situation and mobility factors, other health risk factors, and co-morbidities are taken into account by the case manager. Further non-medical context factors for patient-centered monitoring are, for example, existing responsibilities of the proxy and living wills, religious affiliation, private interests, and relevant biographical events. The Case Management should prevent disruptions between inpatient and outpatient care. It initiates and organizes seamless transitions.
- *Coverage of costs by the health insurance company -* By participating in StroCare, additionally to standard care costs are generated through study nurses, case manager, IT-portal, and neurological outpatient follow-up examinations in the UKE. These costs are paid by the insurance company as well as the standard care costs of neurological rehabilitation .
- *Electronical allocation of capacities (rehabilitation portal) -* Within StroCare an electronic rehabilitation portal will be implemented to speed up and secure a tailored referral to the five cooperating rehabilitation clinics. The portal is also meant to ensure efficient information transfer (medical reports, pictures, diagnostics) (see Fig. 2 for a comparison of the control and the intervention group).

**Fig. 3:**
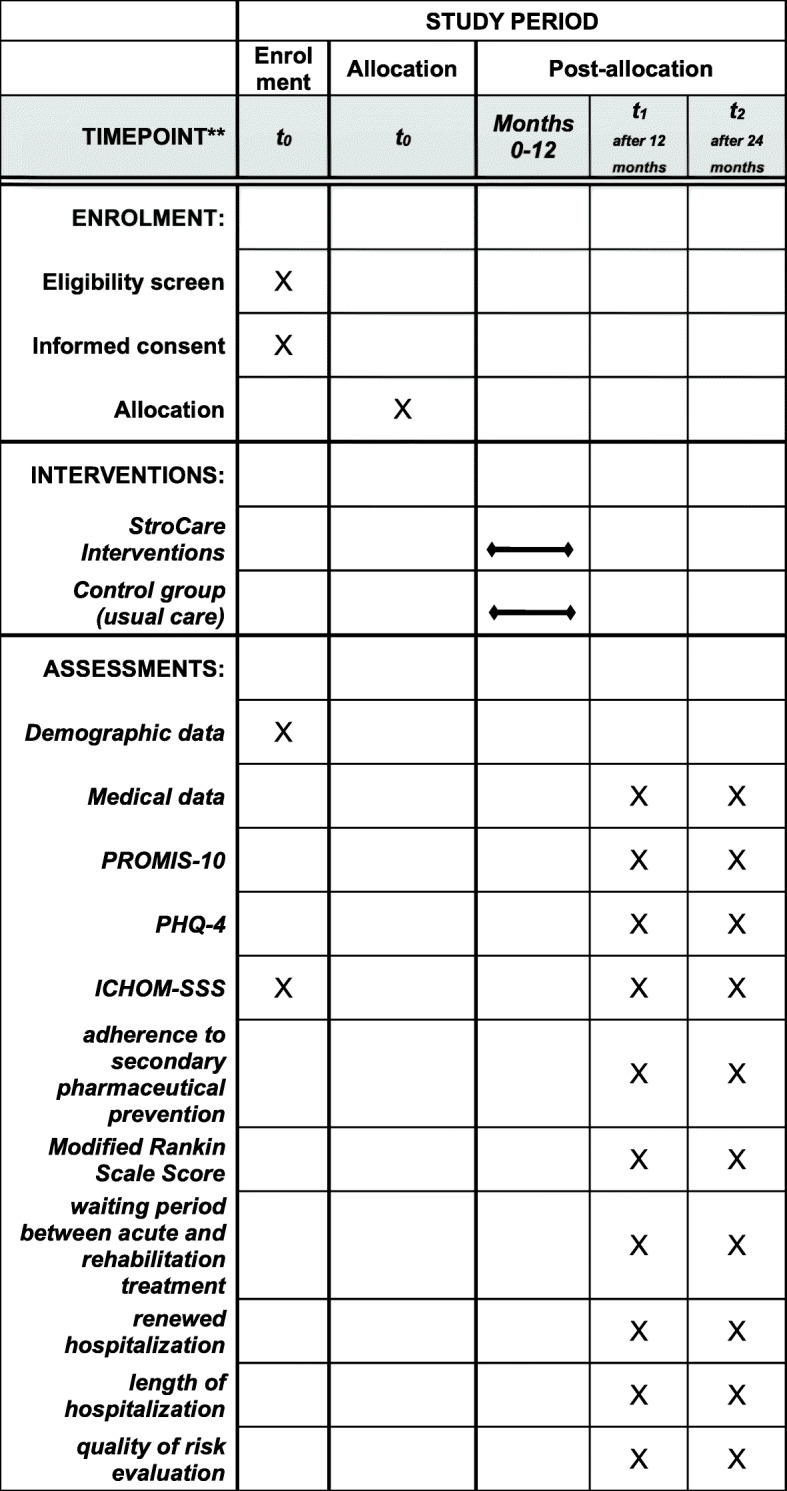
Outcome measures

The portal establishes a network between the acute care hospitals and rehabilitation clinics in order to enable a direct and fast exchange of information (see Fig. 2b). Through the rehabilitation portal a direct inquiry for available capacities can be placed by an employee of the acute hospital to the rehabilitation clinics. Employees of the rehabilitation clinics are able to register when and where vacancies for neurological rehabilitation are available. Once a suitable rehabilitation clinic for the patient is found, the according reports and images can be sent directly to the clinic via the rehabilitation portal. With this software, a seamless transition in treatment is supposed to be achieved, whilst the staff members administrative tasks are reduced.

#### Standard care

Patients allocated to the control group experience the usual stroke care. After initial treatment at an acute clinic, a request for rehabilitation to the health insurance company has to be submitted and approved. Afterwards a suitable clinic for a neurological or geriatric rehabilitation with available capacities has to be found. This may not be achieved immediately after initial stroke treatment by the differently focused acute therapists, and in addition, not every patient receives the rehabilitation treatment best suitable for her/him [3].

### Outcome measures

#### Effectiveness

In order to assess the effect of intervention, patient-reported outcomes will be surveyed measuring health-related quality of life (Fig. 3). They will be measured 12, and 24 months after baseline, using the questionnaire “Patient Reported Outcome Information System 10-Question Short Form” (PROMIS-10). The PROMIS-10 is a standardized questionnaire and consists of two scales measuring mental health and physical health [14], respectively, which will serve as the two primary outcomes. For evaluation of the PROMIS-10, at first a sum score and afterwards a t-score with standardized t-values is calculated. Reference of M ± SD= 50 ± 10 regards to a sample of healthy American subjects.

Additionally, the secondary outcome of depression and anxiety symptoms will be measured at all time points, using the “Patient Health Questionnaire-4” (PHQ-4) [15]. This questionnaire entails the two dimensions, depressive and anxiety symptoms, with two items each. The sum score ranges from 0 to 6 in each dimension. A score of 3 or higher indicates a potential anxiety or depression. The patient’s functional status will be assessed by the modified Rankin Scale questionnaire (smRSq) [16]. The scale of the smRSq ranges from 0 (“no symptoms”) to 6 (“death”). The outcomes of overall survival and stroke recurrence will be measured with the ICHOM-Standard set for Stroke [11]. The ICHOM-Standard Set for Stroke was developed under the coordination of the International Consortium for Health Outcomes Measurements (ICHOM) and measures a range of patient-reported outcomes from functionality to general health status. Further secondary outcomes are: utilization of health care services, patient’s waiting time between treatment phases, success in regaining target values of relevant risk factors (blood pressure, LDL-cholesterol, HbA1c) and costs. These parameters will be measured via the medical patient record and the rehabilitation portal. Also, a sociodemographic questionnaire will be used, measuring age, gender, living situation, partnership, vascular and non-vascular comorbidities.

#### Process evaluation outcomes

With the aim of evaluating the implementation process a qualitative survey will be performed. For conducting qualitative interviews with patients and employees during the intervention phase a semi-structured interview guide will be developed. Five of the indicators proposed by Proctor et al. [17] [17] will be the focus in the interviews and evaluation process acceptability, adoption, appropriateness, feasibility and fidelity. Moreover, to explore the patient-centeredness of the intervention, access to care and clinician-patient relationship will be investigated [18]. Finally satisfaction with treatment will be explored. Interviews will be conducted by scientific staff.

#### Economic evaluation outcomes

Primary economic outcomes are inpatient and outpatient costs, costs for medication and nursery.

### Data analysis

#### Analysis of effectiveness outcomes

The structural equality of the control and intervention groups at baseline will be examined to detect imbalances, using descriptive statistical analysis.

In order to evaluate the intervention effect on the two primary outcomes at the 12-months follow-up, data will be analyzed using linear mixed models. In the fixed part of the model, exposure to the intervention (StroCare vs usual care) will be included as the independent variable of interest. Potential confounding variables will be added as fixed effects in case of baseline imbalance, including demographic characteristics, stroke type and severity of stroke symptoms, as well as vascular and systemic features. The random effects part of the model will include variance parameters for the intercept and for the treatment effect across the participating clinics, respectively. Findings regarding the primary outcomes with an alpha error rate below 0.025 will be considered statistically significant (Bonferroni adjustment for two outcomes).

The primary outcome at the 24-months follow-up and the secondary outcomes will be analyzed using similarly structured models. In the case that data are not normally distributed, generalized mixed models will be applied. Findings regarding the secondary outcomes with an alpha error rate below 0.05 will be considered statistically significant.

Parameter estimates will be reported with 95% confidence intervals. Missing values will be handled using multiple imputation.

#### Analysis of the process outcomes

The audio recordings of the conducted interviews will be transcribed. Qualitative data will be analyzed using qualitative content analysis based on P. Mayring [19]. A deductive-inductive approach will be used with deductive categories derived from literature and inductive derived from the analysis of the qualitative interviews. This method was chosen for its structuring and at the same time adaptable approach.

#### Analysis of the economic outcomes

We will employ both two-part (binomial, gamma) and generalized linear models (gamma distributed outcome) to analyze health care costs, because these models can incorporate the special features of cost distributions – i.e. right skewed distributions that contain a large proportion of zero values [20]. Cox-regression or frailty models as well as generalized linear models will be considered to analyze secondary outcomes such as rehospitalizations, the duration of hospital stays or the utilization of specific services.

### Contacts

Consortium management is executed by the Department of Neurology of the University Medical Center Hamburg-Eppendorf. Recruitment of patients is carried out in cooperation further with two acute care hospitals: Albertinen Krankenhaus and Elbe-Kliniken-Stade-Buxtehude-GmbH. The participating rehabilitation clinics are Reha-Centrum-Hamburg-GmbH, Klinikum-Bad-Bramstedt-GmbH, MediClin-Klinikum-Soltau-GmbH, VAMED-Klinik-Geesthacht and VAMED-Rehaklinik-Damp. Development and execution of the technical intervention is performed by Forcare-GmbH and the department of information technology of UKE. As part of the intervention, the case management will be carried out by the health care insurance BARMER and Lohman & Birkner Medical ServiceCenter GmbH. Evaluation is performed by the Department of Medical Psychology and the Department of Health Economics and Health Care Research at University Medical Center Hamburg-Eppendorf.

### Perspective

This study combines different aspects of health care (primary contact, outpatient managed care, case management, coverage of costs by the health insurance company and an electronical allocation of capacities) in order to improve managed care of stroke patients. The study will not only provide information on the tested intervention but is likely to be helpful for stakeholders and researchers implementing and evaluating complex interventions in stroke care in general. The StroCare interventions will be established at three acute-care clinics with different care structures including five rehabilitation clinics in northern Germany and a nationwide health insurance company. Thus, the health care system will have an evaluated reference model at its disposal for transfer to other regions as well as organizational structures and processes.

## Data Availability

Data will be made anonymous by the research staff during transcription. After completion of the project, the research data/primary data will be stored for another 10 years on durable and secure carriers of the project group in accordance with the proposals for safeguarding good scientific practice of the DFG – German Research Foundation (www.dfg.de). Data evaluation will be carried out by the research staff and no personal data will be published or passed on to third parties. Upon reasonable request that includes a methodologically sound proposal for the usage of data that is also approved by the responsible review committee data may be shared.

## Abbreviations

StroCare: Inter-sectoral, coordinated and evidence-based stroke care
PROMs: Patient-reported outcomes
ICHOM: International Consortium for Health Outcomes Measurements
ICHOM-SSS: ICHOM Standard Set for Stroke
PROMIS-10: Patient Reported Outcome Information System 10-Question Short Form
PHQ-4: Patient Health Questionnaire-4
UKE: University Medical Center of Hamburg-Eppendorf
